# A comprehensive review of management modalities for glaucoma and intraocular hypertension during pregnancy

**DOI:** 10.1007/s10792-026-04030-w

**Published:** 2026-03-25

**Authors:** Nicoleta Anton, Francesca Cristiana Dohotariu, Theodora Armeanu, Răzvan Lisă, Camelia Margareta Bogdănici, Bogdan Doroftei

**Affiliations:** 1 Grigore T. Popa University of Medicine and Pharmacy, University Street, No. 16, 700115 Iași, Romania; 2 Ophthalmology Clinic, Saint Spiridon Emergency Clinic Hospital, Independence Avenue, No. 1, 700111 Iasi, Romania; 3 Origyn Fertility Center, Palace Street No. 3C, 700032 Iasi, Romania

**Keywords:** Pregnancy, Intraocular pressure, Glaucoma surgery, Laser procedures, Minimally invasive glaucoma surgery

## Abstract

**Introduction:**

Glaucoma during pregnancy presents a complex clinical challenge, requiring a careful balance between effective intraocular pressure (IOP) control and fetal safety.

**Background:**

This review examines the ocular changes that occur during pregnancy and their implications for glaucoma progression and management.

**Methods:**

It synthesizes current evidence on IOP fluctuations, disease progression risks, and the safety profiles of pharmacologic, laser, and surgical interventions. Studies were included if they met the following criteria: (1) involved pregnant women diagnosed with glaucoma or ocular hypertension; (2) evaluated medical, laser, or surgical management strategies during pregnancy; and (3) reported outcomes related to intraocular pressure control, optic nerve evaluation, or visual field assessment. Studies focusing exclusively on other ocular complications of pregnancy were excluded. Particular attention is given to emerging trends in minimally invasive glaucoma surgeries (MIGS), diagnostic innovations, and pharmacotherapies tailored to the gestational period. The PRISMA (Preferred Reporting Items for Systematic Reviews and Meta-Analyses) framework was used to guide methodological rigor and transparency.

**Results:**

From 257 initially identified records, 184 studies remained after duplicate removal. Following the title and abstract screening, 73 full text articles were assessed for eligibility. Ultimately, 54 studies met all inclusion criteria and were included in the final review. However, pregnancy introduces diagnostic challenges related to IOP assessment, variability in individual responses, and the possibility of paradoxical IOP elevation. The extent of IOP reduction during pregnancy may be influenced by physiological and demographic factors such as age, parity, systemic blood pressure, and central corneal thickness (CCT), all of which can modulate the degree of IOP change. For instance, multiparous women often exhibit greater IOP decreases than primigravidas. Beta-blockers remain among the most frequently prescribed agents during pregnancy; however, punctal occlusion is recommended to reduce systemic absorption. Alternative treatment options, including selective laser trabeculoplasty (SLT) and minimally invasive glaucoma surgery (MIGS), may be considered based on disease severity and gestational stage.

**Conclusions:**

The findings advocate for a patient-centered approach that integrates evidence-based strategies to preserve maternal vision while ensuring fetal well-being. Ongoing research and interdisciplinary collaboration will be essential in developing new approaches while safeguarding both maternal and fetal health.

## Introduction

Pregnancy induces significant systemic and ocular physiological changes due to hormonal, metabolic, and vascular adaptations. Key gestational hormones–including estrogen, progesterone, human chorionic gonadotropin (hCG), and relaxin–affect aqueous humor dynamics, corneal structure, tear film stability, and intraocular pressure (IOP), with important implications for patients with preexisting glaucoma [1–3].

One of the most consistent ocular changes during pregnancy is a reduction in IOP, typically 2–4 mmHg, particularly in the second and third trimesters. This is mainly due to enhanced uveoscleral outflow, reduced episcleral venous pressure, decreased aqueous humor production, and estrogen-mediated nitric oxide–dependent vasodilation [4, 5].

Corneal changes are also frequently observed. Increased stromal hydration may lead to central corneal thickening, potentially affecting tonometric accuracy. These alterations may also cause transient refractive shifts, reduced contact lens tolerance, and ocular discomfort [6, 7]. Additionally, tear film instability and decreased tear production can contribute to dry eye syndrome, particularly in patients using topical medications with preservatives such as benzalkonium chloride [7, 8].

Various studies have shown that during pregnancy, estrogen can modify CCT, as a result of corneal stromal hydration, which leads to an increase in central corneal thickness and a decrease in measured (but not actual) IOP. In addition, several studies have observed an increase in CCT in pregnant women compared to non-pregnant women, whereas other studies have reported no significant differences between pregnant and non-pregnant women, or no clinically meaningful changes across pregnancy and the postpartum period [8]. In another study, 70 pregnant participants with clinically healthy eyes underwent corneal topographic imaging by Pentacam and biomechanical assessment by Ocular Response Analyzer (ORA) before pregnancy, in the third trimester of pregnancy (34 weeks of pregnancy), and 12 months postpartum. They were compared with age-matched nonpregnant women. The results showed that the differences in corneal tomographic, topographic, and biomechanical parameters before pregnancy, during pregnancy, and postpartum were not statistically or clinically significant [9]. Another study evaluating biomechanical changes in pregnancy showed that a decrease in corneal compensatory intraocular pressure was observed in pregnant women in the third trimester of pregnancy, but without other statistically significant changes resulting from the analysis of the other three parameters (corneal hysteresis, corneal resistance factor and Goldmann correlated intraocular pressure)[10].

Deeper ocular structures are affected as well. Enhanced depth imaging OCT studies have shown choroidal thickening, likely due to increased perfusion. While the clinical impact on glaucoma progression remains uncertain, these changes underscore the need for careful monitoring [11]. Visual field testing may also be less reproducible during pregnancy due to fatigue and mood fluctuations [12].

Pregnancy-related immune modulation, characterized by immunotolerance, may influence neuroinflammatory mechanisms involved in glaucomatous optic neuropathy, although data are limited [13]. Physiological IOP reduction may allow temporary medication reduction or discontinuation in some patients, but close ophthalmologic follow-up and collaboration with obstetric care providers remain essential [14, 15]. Emotional support and clear communication are also critical for alleviating patient anxiety regarding treatment safety [16, 17].

In conclusion, pregnancy induces complex ocular changes that can impact glaucoma management. Understanding these physiological adaptations is essential to optimize maternal visual outcomes throughout gestation [18].

The purpose of this review is to provide an updated overview of intraocular pressure changes during pregnancy and available treatment modalities, with careful consideration of the risks associated with medication use during gestation.

## Materials and methods

A narrative synthesis of the literature was conducted, integrating findings obtained through systematic searches of electronic databases, manual searches, and established scholarly sources.

### Database search strategy

A comprehensive literature review was conducted using several major academic databases, including PubMed, Web of Science, Scopus, Embase, and Google Scholar, to evaluate the relationship between intraocular pressure, glaucoma, and pregnancy [4]. The search was limited to English-language studies published between January 2000 and March 2025. Specific keywords were used, such as “glaucoma AND pregnancy,” “intraocular pressure AND gestation,” “hormones AND ocular pressure,” “ophthalmic medication safety in pregnancy,” “glaucoma surgery AND pregnant patients,” “SLT AND gestational glaucoma,” and “MIGS AND pregnancy.” Boolean operators (AND, OR, NOT) were applied to expand and refine the search results [11]. Data extraction was performed using a standardized template and a qualitative synthesis was conducted to identify key themes related to treatment strategies, medication safety, and clinical outcomes.

To ensure comprehensive coverage, manual searches of the reference lists in key review articles were also performed [2]. The selected articles were managed using citation management software, including EndNote and Mendeley, to remove duplicates and facilitate the organization of studies based on relevance and methodological quality. Additionally, Medical Subject Headings (MeSH) were incorporated into PubMed searches to enhance sensitivity and ensure a more thorough exploration of the relevant scientific literature [17].

### Eligibility criteria

Inclusion criteria. Studies were considered eligible for inclusion if the following criteria were met: (1) the study population included pregnant women diagnosed with glaucoma or ocular hypertension; (2) the study addressed any aspect of glaucoma management during pregnancy, including pharmacological, laser-based, or surgical interventions; and (3) at least one ophthalmologic outcome was reported, such as intraocular pressure control, optic nerve evaluation, or visual field assessment [17].

Exclusion criteria. We excluded studies published in languages other than English, animal or in vitro research, conference abstracts lacking full data, editorials, opinion pieces, and narrative reviews. Additionally, we did not include studies focusing solely on other ocular complications of pregnancy, such as diabetic retinopathy or hypertensive retinopathy, unless they provided comparative or mechanistic insights directly relevant to glaucoma [20]. See Table 1.

**Table 1:** Selection criteria for studies from specialized literature

Inclusion criteria	Exclusion criteria	Keywords
Pregnant women with glaucoma or intraocular hypertension;	Studies published in languages other than English	Glaucoma and pregnancy
Study addressed any aspect of glaucoma management during pregnancy (medication, laser, surgery etc.)	Animal or in vitro research	Intraocular pressure and gestation ophthalmic medication safety in pregnancy
Studies on the evaluation of glaucoma in pregnant women treatment (intraocular pressure control, optic nerve evaluation, or visual field assessment)	Studies focusing solely on other ocular complications of pregnancy (diabetic retinopathy or hypertensive retinopathy etc.)	Glaucoma surgery and pregnant women SLT, MIGS and MICS during pregnancy Trabeculectomy during pregnancy

The selection process was conducted independently by two reviewers with training in ophthalmology and gynecology. An initial screening of article titles and abstracts was followed by full-text assessments of potentially eligible studies. The PRISMA (Preferred Reporting Items for Systematic Reviews and Meta-Analyses) framework was used to enhance transparency and methodological rigor [7].

### Risk of bias assessment

Due to the heterogeneity and small size of most included studies, no formal risk-of bias tool (e.g., ROBINS-I or Cochrane Risk of Bias Tool) was applied. Most studies exhibited a moderate risk of bias, primarily due to small sample sizes, lack of control groups, and potential confounding variables that were not fully addressed. All assessments were conducted manually by the review team. No automated screening tools were used.

## Results

**Fig. 1:**
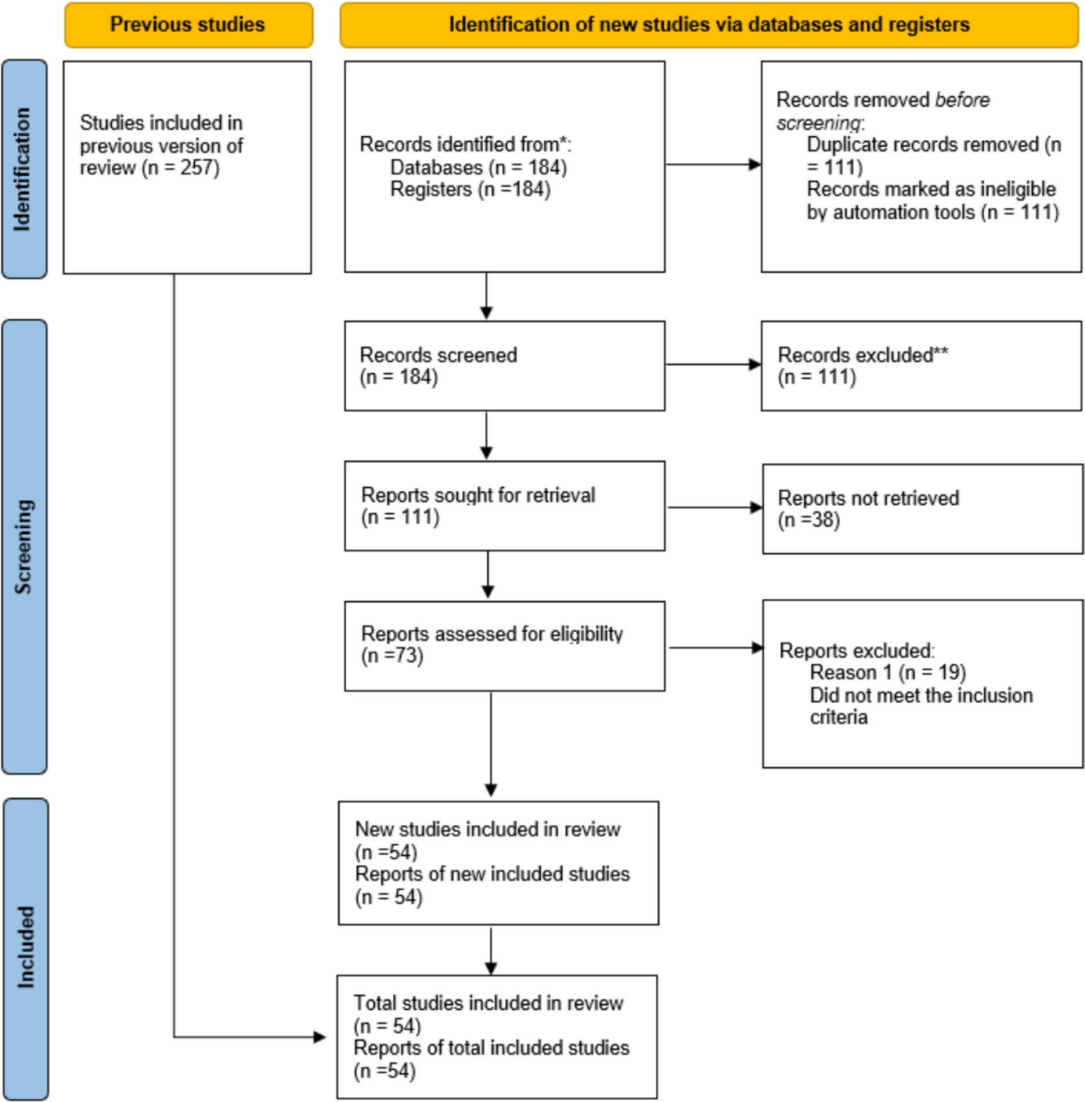
A PRISMA flowchart that was generated to illustrate the study selection process and to increase transparency in reporting

From 257 initially identified records, 184 studies remained after duplicate removal. Following title and abstract screening, 73 full-text articles were assessed for eligibility. Ultimately, 54 studies met all inclusion criteria and were included in the final review. According to studies, although glaucoma during pregnancy is relatively rare, its prevalence among pregnant women is 0.16% in the 15–34 age group and between 0.3 and 0.7% in the 35–44 age group [14]. A PRISMA flow diagram was generated to illustrate the study selection process and enhance transparency in reporting (Fig. 1).

### Changes in intraocular pressure during pregnancy

Intraocular pressure is essential in the diagnosis and management of glaucoma, and it is well established that pregnancy significantly influences IOP levels. A substantial body of evidence indicates a physiological reduction in IOP during pregnancy, particularly in the second and third trimesters [2, 15]. The mechanisms underlying this phenomenon are complex and involve hormonal, vascular, and anatomical changes that alter aqueous humor dynamics.

Among the principal factors contributing to reduced IOP during pregnancy are elevated levels of endogenous estrogen and progesterone [5]. These hormones are believed to affect the trabecular meshwork and uveoscleral outflow pathways. Progesterone, for instance, may reduce aqueous humor production by inhibiting carbonic an-hydrase, while estrogen’s vasodilatory effects are thought to lower episcleral venous pressure, thereby decreasing IOP. Additionally, pregnancy-specific hormone relaxin may promote ocular tissue relaxation and facilitate outflow through non-traditional pathways [11].

Data from Ibraheem et al. (2015) and Wang et al. (2017) support these mechanisms [4, 6]. Their findings indicate that IOP typically decreases by approximately 2–4 mmHg from pre-pregnancy levels, with the most pronounced reductions observed during the second and third trimesters. Postpartum, IOP generally returns to baseline levels. These reductions are more consistently observed in women with normal baseline IOP, whereas those with ocular hypertension or glaucoma may exhibit a less marked response [8].

However, the degree of IOP reduction during pregnancy is not uniform and appears to be influenced by additional physiological and demographic factors. Variables such as age, parity, systemic blood pressure, and CCT can affect the magnitude of IOP change. Multiparous women, for example, often show more substantial IOP reductions compared to primigravidas, possibly reflecting cumulative hormonal effects or adaptive vascular changes [18]. Although diurnal IOP fluctuations persist during pregnancy, their amplitude may be diminished, complicating efforts to accurately monitor disease progression [12]. Salvetat et al. very succinctly describes what mechanisms occur during pregnancy and how pregnancy hormones influence the level of intraocular pressure (as shown in Table 2). The study suggests that these changes are due to pregnancy-related electrolyte changes and elevated levels of estrogen, progesterone, relaxin, and beta-human chorionic gonadotropin, whose concentrations have been shown to be inversely proportional to IOP measurements; moreover, they appear to be associated primarily with increased aqueous flow rather than a negative regulation of water production [18].

**Table 2:** Mechanisms explaining physiological changes in IOP and optic nerve during pregnancy

Variables	Mechanisms	Results
Increased estrogenes	Gestational vasodilation and collagen synthesis may protect RGCs by increasing lamina cribrosa deformability	Increased aqueous production → IOP reduction Optic nerve protection against ischemic damage Increased aqueous trabecular and uveo-scleral outflow → IOP reduction
Increased relaxin	Increased collagen degradation- increased corneal deformability, trabecular permeability, scleral permeability	IOP underestimation Increased aqueous trabecular outflow → IOP reduction Increased aqueous trabecular outflow → IOP reduction
Increased progesterone	Reduces effect of endogenous corticosteroids	Increased aqueous trabecular outflow → IOP reduction
Increased HCG	Increased cyclic adenosine monophosphate in the ciliary body	Reduces aqueous production → IOP reduction
Increased metabolic acidosis	Increased arterial vasodilation → systemic hypotension Increased venous vasodilation → reduces venous pressure	Reduces aqueous production → IOP reduction Increased aqueous trabecular and uveo-scleral outflow → IOP reduction

IOP = intraocular pressure; HCG = human chorionic gonadotropin. Data derived from pertinent literature [18]

Large population studies have found that a greater estrogen life exposure because of early menarche or late menopause and the assumption of post-menopausal hormone replacement therapy (estrogen alone or in combination with progesterone) are significantly associated with a lower risk of POAG. The protective role of estrogen against glaucoma is still under investigation and may be related to different mechanisms, including their vasodilatory effect, with increasing ocular blood flow; the activation of collagen synthesis, which may reduce the deformability of the lamina cribrosa and the optic nerve axon compression at its level; and their neuroprotective effect on the retinal ganglion cells, as demonstrated in animal models [18, 19].

Changes in corneal biomechanics during pregnancy, particularly the increase in central corneal thickness (CCT) due to estrogen-mediated fluid retention, may further affect the interpretation of tonometry measurements. Several studies have reported increased CCT in pregnant women compared with nonpregnant women, particularly during the third trimester. However, the influence of CCT does not fully explain the lower intraocular pressure (IOP) observed in pregnancy. The literature remains inconclusive, as some studies demonstrate an association between increased CCT and IOP changes, whereas others do not, as reflected in current American guidelines. In addition to CCT, other corneal biomechanical parameters relevant to glaucoma, such as corneal hysteresis and corneal resistance factor, have been evaluated. Overall, these parameters do not appear to be significantly affected by pregnancy [8]. Corneal thickening may lead to falsely elevated IOP readings when measured with Goldmann applanation tonometry. Clinicians are therefore advised to interpret such measurements with caution and to consider alternative techniques, such as dynamic contour tonometry or rebound tonometry, which are less influenced by corneal properties. However, a study evaluating corneal biomechanics and IOP changes during pregnancy in a cohort of healthy Chinese women demonstrated increased corneal hysteresis and corneal resistance factor during the second and third trimesters. Notably, the increase in corneal hysteresis was independent of corneal-compensated IOP, suggesting that pregnancy may induce specific alterations in corneal biomechanical properties. These findings may have implications for glaucoma assessment and for the understanding of corneal ectatic disorders during pregnancy. [21].

While a decrease in IOP is typical, a subset of patients may experience paradoxical elevations during pregnancy. This is especially relevant for individuals with narrow-angle glaucoma or those who have undergone prior surgical interventions affecting aqueous outflow. Hormonal and anatomical changes in these patients may promote angle narrowing or exacerbate existing outflow resistance, necessitating close monitoring and, in some cases, therapeutic adjustments [17].

Moreover, systemic complications of pregnancy, such as preeclampsia and gestational hypertension, can also impact ocular health. Preeclampsia, characterized by widespread vascular dysfunction, may contribute to choroidal thickening and, in certain cases, elevated IOP. Although the precise mechanisms are still under investigation, clinicians should remain vigilant for atypical ocular presentations in these high-risk populations [22].

From a clinical standpoint, the pregnancy-associated reduction in IOP presents an opportunity to temporarily reduce or discontinue glaucoma medications, thereby minimizing fetal exposure to potentially teratogenic agents. However, such adjustments must be made cautiously and under close supervision, with frequent monitoring to detect early signs of disease progression. Ancillary diagnostic tools, such as OCT and visual field testing, can provide essential support in assessing disease stability during this period [14].

Regarding IOP changes induced by labor and delivery, the authors have shown that mean IOP increases by approximately 1–2 mmHg during vaginal labor, decreases by 3 mmHg immediately after delivery, and returns to pre-labor levels by 3 days postpartum. Changes in IOP during both vaginal and cesarean delivery are considered not to be clinically significant [18].

In conclusion, pregnancy induces a consistent and often beneficial reduction in IOP through hormonally and vascularly mediated mechanisms. Table 3 provides a concise overview of selected studies that address IOP changes during pregnancy. An individualized approach–incorporating alternative measurement techniques, careful monitoring, and a solid understanding of pregnancy-specific ocular physiology–is crucial for the effective management of glaucoma during this critical period [3].

**Table 3:** Provides a concise overview of selected studies that address intraocular pressure (IOP) changes during pregnancy

Nr	Title	Study type	Number of eyes	Type of treatment	Results	Conclusions
1.	A Narrative Review of the Complex Relationship between Pregnancy and Eye Changes [2]	review	N/A	N/A	Due to hormonal influences, physiological ocular changes during pregnancy have been shown in Caucasian women, so corneal sensitivity, refractive status, intraocular pressure, and visual acuity may change during pregnancy	Periodic ophthalmologic evaluation could detect and treat possible ocular changes early, and the quality of life and visual prognosis of patients should be positive both in the long and short term
2.	Pregnancy hormone to control intraocular pressure? [5]	update	N/A	N/A	For glaucoma patients in pregnancy, though a majority still shows the reduction in IOP, it can increase or remain stable too	Hope that newer targets for glaucoma medications would help us to medically treat uncontrolled glaucoma especially if a surgical option is not feasible
3.	Changes in intraocular pressure and central corneal thickness during pregnancy: a systematic review and Meta-analysis [4]	Meta-Analysis	N/A	N/A	Fifteen studies were included. IOP was significantly decreased during the second and third trimesters of pregnancy	A decrease in IOP is accompanied by an increase in CCT in the second and third trimesters of a normal pregnancy in women
4.	Tear Film Functions and Intraocular Pressure Changes in Pregnancy [6]	Clinical study	270 participants including 165 healthy pregnant women and 105 non-pregnant	N/A	The mean values for IOP (mmHg), TBUT (seconds) and Schirmer’s reading (mm) were: 13.24 ± 2.18, 25.05 ± 9.30, 37.03 ± 17.06 and 14.24 ± 2.66, 22.10 ± 10.81, 50.13 ± 19.10 for cases and controls respectively. Schirmer’s reading (SR) was significantly lower among pregnant women	The implication of this to the policy maker is that appropriate policy should be put in place to encourage routing ocular examination during pregnancy
5.	A Practical Guide to the Pregnant and Breastfeeding Patient with Glaucoma. [8]	review	N/A	N/A	Intraocular Pressure Changes in Pregnant Women with or at Risk for Glaucoma. In a series of 32 ocular hypertensive pregnant women, IOP was noted to be significantly lower than baseline IOP starting at 24 weeks gestation, with an average IOP lowering of 24%, most of this manifesting between the 24th and 30th week of gestation	These patients, have a moderate risk of IOP elevation and disease progression during pregnancy and should be followed carefully (i.e., every 1–3 months) Patients with a family history of IOP should be monitored, as studies show they may develop increased pressure in the second trimester
6.	Management of Glaucoma in Pregnancy [12]	review	N/A	N/A	Our review suggests that individually, intraocular pressure is lower in a pregnant woman when compared with a nonpregnant woman	An open dialog and close relationship between clinician and females of child-bearing age about the risks and options of pregnancy in glaucoma is vital to producing best possible outcomes for all involved
7.	Is Estrogen a Therapeutic Target for Glaucoma? [13]	review	N/A	N/A	Increased estrogen states may confer a reduced risk of glaucoma or glaucoma-related traits such as reduced IOP. Pregnancy, a hyperestrogenism state, is associated with decreased IOP during the third trimester	Increasing evidence suggests that lifetime exposure to estrogen may alter the pathogenesis of glaucoma. Estrogen exposure may have a neuroprotective effect on the progression of POAG but further studies need to confirm this finding

Although there are few studies that associate intraocular hypertension with pregnancy, the American Academy of Ophthalmology guidelines address the management of intraocular hypertension in pregnancy, discuss possible mechanisms of IOP changes, and discuss behaviors and medical treatments that may influence IOP during pregnancy. Also, patients with a family history of glaucoma may develop IOP during pregnancy in the second trimester, and therefore should be monitored carefully (every 1–3 months), presenting a case that developed elevated IOP that required bilateral surgery [8].

### Glaucoma progression and management during pregnancy

Although pregnancy is often associated with a physiological reduction in IOP, its influence on glaucoma progression is more complex. For women with moderate to severe glaucoma or a history of rapid disease progression, pregnancy may paradoxically represent a period of increased risk due to hormonal and vascular changes that disrupt ocular homeostasis [15, 18].

While relatively rare, glaucoma progression during pregnancy has been documented in case series and retrospective analyses. A notable study by Mathew et al. (2019) reported cases in which patients who discontinued their medications early in pregnancy–primarily due to concerns about fetal safety–subsequently experienced measurable optic nerve damage and visual field deterioration [12]. These findings underscore the importance of rigorous monitoring throughout pregnancy, even when IOP readings appear stable. It is hypothesized that glaucomatous damage may continue despite modest IOP reductions if optic nerve perfusion or neuroprotective mechanisms are compromised [13].

Several physiological changes during pregnancy may affect optic nerve integrity independently of IOP. Hormonal fluctuations, increased blood volume, altered vascular resistance, and systemic fluid retention can collectively influence ocular perfusion pressure, potentially impairing nutrient delivery to retinal ganglion cells [3]. Some researchers propose that the widespread vasodilation observed in pregnancy could paradoxically result in relative hypoperfusion of the optic nerve head, particularly in eyes already prone to vascular dysregulation [20].

Behavioral and psychological factors also influence disease management during pregnancy. Anxiety, fatigue, and the demands of prenatal care may reduce adherence to ophthalmic follow-up visits, medication regimens, and self-monitoring routines [14] Missed appointments or inconsistent medication use can compromise disease control. Therefore, patient education and proactive counseling are essential, helping patients prioritize ocular health alongside prenatal responsibilities.

Effective management of glaucoma during pregnancy requires an individualized risk–benefit assessment for each patient. Key considerations include current disease se-verity, IOP control, treatment history, and the availability of non-pharmacologic alter-natives such as laser therapy or minimally invasive glaucoma surgeries (MIGS) [8]. These factors guide the development of a personalized care plan tailored to each patient’s needs. Cross-disciplinary collaboration between ophthalmologists and obstetricians becomes particularly critical when treatment intensification is necessary. A coordinated care approach helps safeguard both maternal vision and fetal well-being [16].

Preconception counseling is an emerging and valuable strategy for women with glaucoma planning pregnancy. These consultations offer an opportunity to optimize disease control, transition to safer therapies where appropriate, and establish a multi-disciplinary care framework before conception [17]. Early discussions also facilitate contingency planning if surgical intervention becomes necessary.

Although visual field-testing during pregnancy may be affected by fatigue and reduced attention, it remains an important tool. Imaging of the retinal nerve fiber layer and optic nerve head using optical coherence tomography (OCT) is also recommended to aid in the early detection of disease progression [23].

Overall, managing glaucoma during pregnancy involves balancing disease stability with the safety of both mother and child. Clinical evaluations, OCT imaging, and cautious IOP monitoring should be conducted regularly, with management strategies reevaluated each trimester to account for changing risks. The postpartum period also warrants close attention, as hormonal normalization and IOP rebound may reveal previously undetected disease progression [24].

In summary, although glaucoma progression during pregnancy is relatively uncommon, it remains a significant clinical concern–particularly in high-risk patients. A proactive, flexible, and patient-centered approach–grounded in regular monitoring and interdisciplinary collaboration–can help ensure favorable outcomes for both mother and baby [25]. In Table 4 we present a concise overview of studies addressing both medical and surgical management of glaucoma.

**Table 4:** Presents a concise overview of studies addressing both medical and surgical management of glaucoma

Nr	Title	Study type	Number of patients/ eyes	Type of treatment	Results	Conclusions
1.	A Practical Guide to the Pregnant and Breastfeeding Patient with Glaucoma. Ophthalmol Glaucoma [8]	Update	N/A	N/A	FDA: category A, deemed safe; category B, possibly safe to use in pregnancy; category C, adverse effects reported in animal studies; category D, definite risks but possible benefits; and category X, drugs with known risks to the fetus that cannot be outweighed by possible benefits Most glaucoma medications in this classification system are category C	Safe medical, laser, and surgical management options may be available for carefully selected pregnant patients with glaucoma
2.	Pregnancy outcomes in the medical management of glaucoma: An interna-tional multicenter descriptive survey [16]	Clinical case	114 pregnancies of 56 patients (mean 2.0 pregnancies per patient) were included	Of the 111 pregnancies, 20 (18.0%) used no medications and 91 (82.0%) used at least one medication. Medications were topical carbonic anhydrase inhibitors (n = 45), beta-blockers (n = 55), alpha-agonists (n = 56), and prostaglandin analogues (n = 28)	Reported outcomes included preterm contractions or labor (6.3%), miscarriage (4.5%), stillbirth (4.5%), induction of labor (11.9%), emergency or unplanned cesarean delivery (13.9%), NICU admission (15.8%), congenital anomalies (8.1%), and low birth weight (10.9%). Most NICU admissions associated with alpha-agonist exposure occurred following third-trimester use.”	Topical antiglaucoma medications are generally considered safe during pregnancy; however, third-trimester exposure to alpha-agonists may be associated with an increased risk of neonatal intensive care unit (NICU) admission
3.	Glaucoma Surgery in Pregnancy: A Case Series and Literature Review [17]	Case series	6 eyes of 3 pregnant patients with uncontrolled glaucoma using maximum tolerable medications	All 3 patients had juvenile open-angle glaucoma and were on various anti-glaucoma medications, including oral acetazolamide. The first case described underwent trabeculectomy without antimetabolites in both eyes. The second patient had an Ahmed valve implantation in both eyes during the second and third trimesters. The third case had a Baerveldt valve implantation under general anesthesia in the second trimester	Case 1. The IOP was 13 mm Hg_2_ weeks after the second operation in both eyes and then was stable at low teens throughout pregnancy. She gave birth to a normal baby at the 38th week of gestation. The baby weighed 3050 g and her Apgar score was 10 case 2. The IOP was 18 mm Hg in both eyes with dorzolamide over the last 2 weeks of pregnancy and 2 months after delivery. The mother gave birth to a healthy baby with a birth weight of 2750 g and an Apgar score of 9. Case 3. One month later, the IOP was 14 mm Hg in the right eye and 16 mm Hg in the left eye. The patient delivered a healthy baby girl with a birth weight of 2523 g at term with an Apgar score of 10	In selected pregnant patients with glaucoma and uncontrolled IOP threatening vision, incisional surgery may achieve favorable maternal outcomes without significant fetal risk
4.	Glaucoma medications in pregnancy [26]	Review	N/A	N/A	Category A: Safety established using human studies Category B: Presumed safety based on animal studies, but no human studies Category C: Uncertain safety, with no human studies and animal studies showing adverse effect Category D: Unsafe; evidence of risk that in certain clinical circumstances may be justifiable Category X: Definitely unsafe, with the risk of use outweighing any possible benefit	No topical antiglaucoma agents have demonstrated strong evidence of fetal safety in human studies Alternative IOP-lowering strategies, including surgical interventions, may be considered prior to pregnancy

Therefore, the conclusions drawn from the studies are that no topical antiglaucoma agent has strong evidence of safety for the fetus based on human studies.

#### Medical treatment of glaucoma during pregnancy

The medical management of glaucoma during pregnancy requires an individualized approach aimed at achieving adequate intraocular pressure (IOP) control while minimizing fetal risk [11]. The safety profile of antiglaucoma medications during pregnancy varies considerably, and available evidence is often limited or derived primarily from animal studies.

Beta-adrenergic blockers (FDA Category C), such as timolol, may be associated with fetal bradycardia and neonatal respiratory depression, particularly when used in late pregnancy. Nevertheless, they are commonly prescribed, often in conjunction with punctal occlusion to reduce systemic absorption [8, 14]. Non-selective topical beta-blockers are contraindicated in patients with asthma, chronic obstructive pulmonary disease (including emphysema and chronic bronchitis), certain cases of congestive heart failure, bradycardia, and heart block. Neonatal symptoms related to beta-blockade are generally mild and typically resolve within 48h [14, 15].

Alpha-adrenergic agonists, particularly brimonidine (Category B), are considered relatively safe during early pregnancy but should be discontinued prior to delivery because of the risk of neonatal central nervous system depression [14, 15]. Apraclonidine is used less frequently due to lower efficacy and a higher incidence of adverse effects, including dry mouth, drowsiness, and tachyarrhythmias. [14–16].

Carbonic anhydrase inhibitors (CAIs) are classified as Category C. Topical formulations are preferred over systemic agents, as oral acetazolamide has been associated with fetal skeletal abnormalities in animal studies. Systemic administration is generally avoided during the first trimester unless clearly indicated [17, 27]. Prostaglandin analogs (Category C) are typically avoided because of their theoretical potential to induce uterine contractions; their use is generally reserved for refractory cases. [28, 29]

Cholinergic agents such as pilocarpine are rarely used during pregnancy due to their adverse effect profile and the lack of robust safety data. These agents, also classified as Category C, should be considered only when safer alternatives are unavailable [15].

Netarsudil (Rhopressa), a Rho kinase inhibitor, is a newer antiglaucoma agent without an assigned FDA pregnancy category. Although animal studies have not demonstrated clear teratogenic effects, the multiple downstream targets of Rho kinase inhibition raise theoretical concerns regarding potential adverse fetal outcomes. [14–16].

Hyperosmotic agents (mannitol, urea, isosorbide, and glycerol) induce hyperosmolarity within the intravascular compartment and are used almost exclusively in emergency situations to manage acute IOP elevation, particularly during primary or secondary angle-closure glaucoma attacks. These agents are classified as Category C. No well-controlled animal or human studies evaluating their use during pregnancy are available; therefore, their administration should be approached with caution [2, 8, 16, 18].

To minimize systemic drug absorption, several strategies can be employed. Techniques such as punctal occlusion for two to three minutes after instillation and eyelid closure can significantly reduce systemic exposure [12]. Additional measures include using the lowest effective dose, minimizing dosing frequency, and opting for preservative-free formulations when available, to reduce ocular surface irritation and systemic absorption [30]

In selected cases, it may be appropriate to temporarily suspend pharmacologic therapy during pregnancy. In such situations, alternative treatments such as selective laser trabeculoplasty (SLT) or minimally invasive glaucoma surgery (MIGS) may be considered, depending on the severity of the disease and gestational timing. Table 5 summarizes the main antiglaucoma drug classes and their considerations during pregnancy.

**Table 5:** Summary of common glaucoma medications and their use in pregnancy

Drug class	Example agents	FDA pregnancy category	Side effects	Contraindications
Beta-blockers	Timolol, Betaxolol	C	Use with caution, avoid near delivery, may result in fetal and neonatal bradycardia, arrhythmia, hypotension and hypoglycaemia	Severe, symptomatic bradycardia, cardiogenic shock, and third-degree heart block, along with severe asthma, uncontrolled heart failure, and severe peripheral arterial disease
Alpha-agonists	Brimonidine	B	Safe early on, discontinue before delivery Systemic circulatory effects include dry mouth, drowsiness and tachyarrhythmias	patients with a history of significant cardiac, respiratory, or liver problems should avoid them
CAIs (topical)	Dorzolamide, Brinzolamide	C	Use if benefits outweigh risks. For systemic CAIs, major side effects include paresthesia, malaise, gastrointestinal disturbances, renal disorder, blood dyscrasia, and metabolic acidosis	Low corneal endothelial cell count
CAIs (oral)	Acetazolamide	C	Avoid in first trimester Use of acetazolamide in late pregnancy has been statistically associated with neonatal electrolyte imbalance and metabolic acidosis	Hypersensitivity to sulfonamides (sulfa allergy) Marked kidney disease or severe renal impairment Severe liver disease or cirrhosis Hypokalemia Hyponatremia Hyperchloremic metabolic acidosis Respiratory acidosis
Prostaglandins	Latanoprost	C	Avoid due to uterine effects Systemic: dyspnea, chest pain/angina, exacerbation of asthma	
Cholinergics	Pilocarpine	C	Rarely used, limited data Systemic effect: intestinal cramps, bronchospasm, headache	Uncontrolled Asthma Severe Cardiovascular Disease: bradycardia, heart block, or myocardial infarction
Rho kinase inhibitor	Netarsudil (Rhopressa)	C	Avoid during pregnancies, limited data Systemic: headache, nasal discomfort, dermatitis allergic, polycondritis, escoriation	
Hyperosmotic agents	mannitol, urea, isosorbide, and glycerol	C	Avoid, increase diuresis, hyponatremia, renal failure, cerebral edema, headache,blurred vision, convulsions, nausea vomiting, dehydration, heart failure, hypotension, and tachycardia No animal or human studies about the use of these drugs during pregnancy are available	

Close collaboration between ophthalmologists and obstetricians is essential for managing glaucoma patients who require ongoing treatment during pregnancy. Doctors’ decision-making together with the patient is crucial to developing an effective and acceptable treatment plan. Preconception counseling is also highly recommended, as it allows for the adjustment of medications and the exploration of safer therapeutic alternatives prior to conception [31–33].

In conclusion, the medical management of glaucoma during pregnancy requires navigating a narrow therapeutic window. Although several medications can be used with caution, successful outcomes depend on minimizing systemic exposure, implementing close monitoring, and considering non-pharmacologic therapies such as selective laser trabeculoplasty (SLT) or minimally invasive glaucoma surgery (MIGS) when appropriate [25].

#### Selective Laser Trabeculoplasty (SLT) during pregnancy

Selective laser trabeculoplasty (SLT) has become a particularly valuable treatment modality for pregnant women with glaucoma, especially for those who are unable to continue or initiate pharmacologic therapies due to concerns about fetal safety [11, 33]. As a noninvasive, outpatient procedure with a favorable safety profile, SLT offers several advantages within the unique physiological context of pregnancy.

SLT lowers intraocular pressure (IOP) by targeting pigmented cells within the trabecular meshwork using low-energy laser pulses, thereby enhancing aqueous humor out-flow through the conventional drainage pathway [30]. Importantly, this mechanism avoids the systemic absorption associated with topical and oral glaucoma medications, making SLT an especially attractive option during pregnancy [14].

One of the primary benefits of SLT in pregnant patients is the elimination of systemic drug exposure, thereby minimizing potential teratogenic risks [13]. Since the procedure acts locally at the trabecular meshwork without introducing pharmacologic agents into systemic circulation, the risk of adverse fetal outcomes is negligible. Consequently, SLT is often considered either as a first-line therapy or as an adjunctive measure for patients seeking to avoid fetal drug exposure.

Although the available evidence is largely limited to case reports and small observational studies, outcomes of selective laser trabeculoplasty (SLT) during pregnancy have been encouraging. Studies by Výborný (2017) and Strelow and Fleischman (2020) report effective intraocular pressure (IOP) reduction, good tolerability, and no observed maternal or fetal complications [15, 35]. SLT has also been performed during preconception planning to minimize exposure to antiglaucoma medications. Particularly, Výborný (2017) demonstrated that bilateral SLT achieved adequate IOP control without adjunctive medical therapy during pregnancy and for up to six months postpartum in women with primary open-angle glaucoma. Based on the available data, SLT appears to represent a promising and potentially safe therapeutic option when performed by experienced clinicians [35].

Certain practical considerations must be addressed when planning SLT during pregnancy. The procedure is typically performed in a sitting position, which is generally well tolerated in early gestation [25].In later stages of pregnancy, modifications may be necessary to accommodate maternal comfort and prevent inferior vena cava compression. Additionally, although SLT usually does not require systemic anesthesia, any topical anesthetic agents used should be selected with careful consideration of their safety during pregnancy [30].

Postoperative care following SLT is minimal. Unlike filtering surgeries, SLT does not require intensive postoperative management or the use of corticosteroids, further enhancing its appeal during pregnancy [8]. Temporary use of non-steroidal anti-inflammatory drugs (NSAIDs) may be employed to address transient inflammation, but clinicians often prefer preservative-free, pregnancy-safe formulations and limit their use to short durations.

Another advantage of SLT is its repeatability; if the IOP-lowering effect diminishes over time, the procedure can be safely repeated. This makes SLT an excellent option for long-term management, particularly in women planning multiple pregnancies or seeking to minimize systemic drug exposure [36, 37]. Some clinicians even advocate for preconception SLT to optimize IOP control before pregnancy.

Patient selection for SLT should be based on factors such as open-angle anatomy, mild to moderate IOP elevation, and intolerance or contraindication to medical therapy [2, 38]. Its effectiveness in pregnant patients appears comparable to that observed in the general population, with similarly high success rates and minimal complications. However, careful individualized assessment remains essential.

SLT represents an effective and safe treatment option for glaucoma management during pregnancy. Its non-invasive nature, lack of systemic drug exposure, and well-documented efficacy make it particularly well-suited to this unique patient population. While larger, high-quality studies are needed to confirm long-term outcomes, current evidence supports the integration of SLT into the glaucoma treatment algorithm for pregnant patients.

#### Minimally Invasive Glaucoma Surgery (MIGS and MICS) during pregnancy

Minimally Invasive Glaucoma Surgery (MIGS) and Minimally Invasive Conjunctival Surgery (MICS) represent significant advancements in the surgical management of glaucoma, offering fewer invasive alternatives to traditional filtering procedures. These techniques are characterized by smaller incisions, minimal tissue disruption, faster recovery times, and lower complication rates–qualities that are especially advantageous during pregnancy, when minimizing surgical risk and systemic exposure is paramount [18].

The use of anesthetics in pregnant women is performed only when absolutely necessary and in the lowest amount and concentration adapted to the interventions performed. Most local anesthetics used (lidocaine, prilocaine, and etidocaine are classified as Category B according to historical FDA pregnancy categories) had no teratogenic effects. Bupivacaine and mepivacaine can cause fetal bradycardia. These are drugs that belong to the FDA class C in animal studies and can cause teratogenic effects. [2, 17].

Several MIGS procedures have been successfully performed in pregnant patients, particularly when pharmacological and laser therapies have proven inadequate. Devices such as the Xen Gel Stent, iStent, and Hydrus Microstent, as well as procedures like the Kahook Dual Blade goniotomy, have yielded favorable outcomes, although current data primarily originate from case reports and small case series [30, 36].

For instance, Zehavi-Dorin et al. (2019) reported successful bilateral implantation of the Xen Gel Stent in a pregnant patient with early-onset primary open-angle glaucoma, achieving significant IOP reduction without adverse pregnancy outcomes [39–41].

MIGS offers an appealing safety profile, largely due to its ab interno approach, which preserves conjunctival tissue and reduces the risk of postoperative complications such as bleb leaks. In ab interno approaches, the use of antimetabolites such as mitomycin C is generally unnecessary. This is particularly relevant during pregnancy, as mitomycin C is classified as FDA Category D, indicating positive evidence of human fetal risk based on adverse reaction data or human studies [38, 39]. Avoiding such agents may therefore represent an advantage in pregnant patients. Many minimally invasive glaucoma surgery (MIGS) procedures can be performed under local or topical anesthesia, thereby reducing fetal exposure to systemic anesthetic agents [43–45]

MICS, although less widely adopted, represents an innovative extension of minimally invasive techniques. By completely avoiding conjunctival incisions, MICS may promote faster postoperative recovery and facilitate intraocular pressure (IOP) control independent of subconjunctival filtration blebs. Emerging evidence suggests that MICS may offer a potentially safer surgical option for selected pregnant patients, particularly those with advanced or refractory disease [34, 46].

Surgical decision-making regarding the use of MIGS or MICS during pregnancy must consider disease severity, gestational age, prior treatment responses, and patient preferences. A comprehensive, multidisciplinary approach involving ophthalmologists, obstetricians, and anesthesiologists is essential to optimize outcomes [47, 48].

Postoperatively, MIGS procedures typically require fewer medications and less intensive follow-up than conventional trabeculectomy–an advantage during pregnancy, when patient mobility and access to care may be limited [15]. Moreover, MIGS often reduces or eliminates the need for prolonged corticosteroid therapy, further minimizing systemic exposure.

Nevertheless, it is important to recognize that the evidence supporting the use of MIGS and MICS during pregnancy remains preliminary and is based largely on case reports [16]. In Table 6 we summarize the advantages of MIGS versus MICS during pregnancy. While early outcomes are encouraging, these procedures should be used with caution and reserved for cases in which conservative therapies have failed.

**Table 6:** Summarizes the advantages of MIGS versus MICS during pregnancy

Procedure	Advantages	Disadvantages	Use in pregnancy
MIGS	Safe Ab interno approach Low risk of postoperative complications (e.g., conjunctival leakage) Can be performed under local or topical anesthesia (avoiding fetal exposure to systemic agents)	May require antimetabolites like mitomycin C, whose safety in pregnancy is unconfirmed	Using local or topical anesthesia avoids fetal exposure to systemic agents
MICS	Promotes faster healing Bleb independent IOP control Avoids conjunctival incisions	Same advanced or refractory disease	Surgical options are safer for selected pregnant patients

In conclusion, MIGS and MICS represent promising minimally invasive surgical alternatives for the management of glaucoma during pregnancy. Their favorable safety profiles, compatibility with local anesthesia, and reduced postoperative requirements make them attractive options for selected pregnant patients requiring surgical intervention. Ongoing research is essential to fully establish their efficacy and safety in this unique patient population.

#### Surgical treatment

Trabeculectomy During Pregnancy. Trabeculectomy remains a cornerstone surgical option for the management of glaucoma, typically reserved for moderate to severe cases unresponsive to medical or laser interventions. In the context of pregnancy, however, the decision to proceed with trabeculectomy requires a careful risk–benefit analysis, weighing potential risks to both maternal and fetal health.

A major concern with trabeculectomy during pregnancy is the use of adjunctive anti-metabolites such as mitomycin C (MMC) and 5-fluorouracil (5-FU). Although these agents enhance surgical success by reducing postoperative fibrosis and bleb failure, their potential for systemic absorption and teratogenicity presents significant concerns. Neither MMC nor 5-FU is FDA-approved for use during pregnancy, and animal studies suggest embryotoxic effects. Consequently, their use is generally avoided or applied with caution, typically during the second trimester when organogenesis is complete and surgical intervention is deemed necessary [17, 30].

Clinical evidence–primarily from case reports and small series–indicates that trabeculectomy can be performed safely during pregnancy using local anesthesia to minimize fetal exposure. For example, Banad et al. (2020) reported successful outcomes in pregnant patients who underwent trabeculectomy with modified techniques minimizing or omitting antimetabolite use [36]. In some cases, safer adjunctive alternatives such as collagen matrix implants (e.g., Ologen) have been employed to support surgical outcomes without the risks associated with conventional antimetabolites [44].

The choice of anesthesia is critical. Local techniques, including peribulbar and sub-Tenon’s blocks, are preferred due to their minimal systemic absorption and low fetal risk. Lidocaine, classified as an FDA Category B drug, is generally considered safe during pregnancy. General anesthesia should be reserved for cases where local anesthesia is inadequate and must be administered under multidisciplinary oversight involving anesthesiologists and maternal–fetal medicine specialists [25, 26, 45].

While the core steps of trabeculectomy remain consistent, procedural modifications are recommended during pregnancy. These include optimizing maternal positioning to avoid inferior vena cava compression, minimizing surgical time, and selecting intraoperative agents with favorable safety profiles. Gentle tissue handling and the use of absorbable sutures are advised to reduce postoperative inflammation and limit the need for prolonged corticosteroid therapy, thereby minimizing systemic exposure [11].

Postoperative management requires careful monitoring. Although corticosteroids are essential for controlling inflammation, shortened treatment regimens or substitution with non-steroidal anti-inflammatory drugs (NSAIDs) may be appropriate. Regular follow-up is essential to monitor bleb function, IOP stability, and wound healing. Non-invasive imaging modalities such as anterior segment optical coherence tomography (OCT) offer a safe and effective means of assessing bleb morphology without risk to the fetus [12, 47, 48]

Regarding surgical timing, the second trimester is generally regarded as the safest period for elective ocular surgery, as it avoids the higher risk of spontaneous abortion in the first trimester and preterm labor in the third. However, urgent intervention may be required at any stage if there is rapid IOP elevation or progressive optic nerve damage [41, 49].

Psychological support and comprehensive informed consent are crucial, especially given the heightened anxiety many pregnant patients experience concerning fetal risks. Thorough counseling, clear communication of both known and potential risks, and shared decision-making are key elements of ethical, patient-centered management [8, 50, 51].

Although trabeculectomy during pregnancy poses distinct challenges, it can be performed safely and effectively when conducted under carefully controlled conditions. For patients at significant risk of vision loss who cannot be managed adequately with medical or laser therapies, trabeculectomy remains a vital surgical option [5, 49]. With meticulous planning, judicious anesthesia use, and multidisciplinary cooordination, favorable maternal and fetal outcomes can be successfully achieved [18, 53]. Table 7 summarizes the advantages and pregnancy-specific considerations of trabeculectomy.

**Table 7:** Summary of trabeculectomy advantages and pregnancy considerations

Procedure	Advantage	Anesthesia	Precautions
Trabeculectomy	Can be performed safely during pregnancy	Local anesthesia (e.g., lidocaine), classified as FDA Category B and generally considered safe for use during pregnancy	Thorough counseling Clear communication Comprehensive informed consent
	Remains a vital surgical option for advanced cases	General anesthesia (used only when local anesthesia is insufficient), under multidisciplinary supervision	

In general, the therapeutic trimester plan of pregnancy and the complications that may occur must be known; therefore, the table below describes the drugs and related studies that can be administered during preconception. The administration of topical anti-glaucoma medications during pregnancy is highly dependent on the stage of gestation. Patients should be thoroughly counseled about the potential risks of anti-glaucoma pharmacotherapy during pregnancy and the possible need for laser or surgical intervention prior to conception. Table 8 describes in detail the treatment methods in the preconception, conception, labor, and postpartum periods. [2, 7, 8, 12, 16, 17].

**Table 8:** Drugs and related studies that can be administered during preconception, conception, labor and postpartum period

Stages of pregnancy	Management	Observations
Pre-Conception	Informing the patient about the risks of antiglaucoma medication Perform laser or surgical treatment before pregnancy	Glaucoma treatment planning should begin before pregnancy
First trimester	Stop antiglaucoma medication Brimonidine, a Category B drug, may be the safest option for the first trimester/ punctal occlusion recommended Surgery is not recommended	The first 8 weeks are most critical for major organogenesis Anesthetics, sedatives, and antimetabolites used in glaucoma surgery may be teratogenic ALT or SLT is an alternative glaucoma treatment safe in all trimesters
Second trimester	Brimonidine continues to be considered the first-line agent Beta blockers can be added only if they are absolutely necessary, careful monitoring Prostaglandin analogues are third-line-they are associated with a risk of premature birth	Beta-blockers can be used with regular fetal heart rate monitoring Fetal growth should be monitored for potential restriction ALT or SLT is an alternative glaucoma treatment that can be performed in all trimesters
Third trimester	Brimonidine, beta-blocker, or topical carbonic anhydrase inhibitors, can be used with caution Latanoprostene bunod and netarsudil have a theoretical risk of stalled labor Glaucoma surgery can be performed with caution in second and third trimester	ALT or SLT is an alternative glaucoma treatment that can be performed in all trimesters
Labor	There is no evidence to support recommending Caesarean section (C-section) or elective pregnancy termination specifically for glaucoma For patients in the early postoperative period after tube surgery or trabeculectomy, C-section should be discussed with the obstetrician	Normal vaginal labor has not been shown to alter IOP in healthy women C-section may be preferred in advanced glaucomatous damage or trabeculectomy-operated patients In patients with prior filtering surgery, the risk of progression or bleb-related complications should be discussed jointly with the obstetrician
Postpartum	Brimonidine is contraindicated in lactating mothers due to risk of CNS depression in the newborn Betablockers are carefully administered to newborns with congenital heart disease	The lowest effect dose of these medications should be considered when used in the breastfeeding period

Brimonidine is the only antiglaucoma medication classified as FDA pregnancy category B. Studies in pregnant rats and rabbits have shown that brimonidine administered at very high doses, approximately 120–190 times the therapeutic dose in humans, caused maternal toxicity, but did not affect fertility or cause fetal malformations. In conclusion, as a general recommendation, antiglaucoma therapy is indicated only for pregnant or lactating patients with moderate/advanced glaucoma, who are at risk of developing functional impairment or diminished vision-related quality of life, or when the risks of progressive disease may outweigh the potential side effects of the treatment itself [2, 18]

### Prevalence and subtypes of glaucoma in pregnant and lactating women

The precise incidence and prevalence of glaucoma during pregnancy and lactation remain undetermined. Epidemiological data suggest that among women of childbearing age, the overall prevalence of glaucoma is approximately 1%, with primary open-angle glaucoma (POAG)–the most common subtype–affecting roughly 0.5% of this population. Although traditionally considered uncommon in reproductive-age women, glaucoma during pregnancy and lactation is increasingly recognized as a clinically relevant condition. In a national survey of 282 ophthalmologists conducted in the United Kingdom in 2007, more than one-quarter of respondents reported prior experience managing glaucoma in pregnant or breastfeeding patients [54].

Given that POAG and primary angle-closure glaucoma (PACG) predominantly manifest after the age of 60 [1], glaucoma diagnosed in women of reproductive age is generally attributable to less common subtypes. These include congenital glaucoma; glaucoma associated with anterior segment dysgenesis (e.g., Axenfeld-Rieger syndrome, Sturge-Weber syndrome, Peters anomaly, aniridia, and iridocorneal endothelial [ICE] syndrome); juvenile open-angle glaucoma (JOAG); and secondary glaucoma resulting from uveitis, ocular trauma, diabetes mellitus, or postoperative complications (e.g., following penetrating keratoplasty or vitreoretinal surgery). Glaucoma associated with pseudoexfoliation or pigment dispersion syndromes may also occur in this age group [55]. Data regarding glaucoma prevalence before the age of 40, particularly in women of childbearing age, remain limited [56]. In a Japanese population-based study, the prevalence of open-angle glaucoma–defined by characteristic visual field defects with corresponding optic nerve findings–was 0.48%, 0.42%, and 0.73% among women aged 15–24, 25–34, and 35–44 years, respectively.

Secondary inflammatory glaucoma during pregnancy presents additional challenges, as hormonal and immunologic changes may exacerbate intraocular inflammation and elevate intraocular pressure (IOP). Management requires a careful balance between effective IOP and inflammation control and fetal safety, favoring agents and interventions with more established safety profiles. Notably, these subtypes are frequently refractory to standard therapeutic approaches [11 further complicating management during pregnancy and lactation, when both maternal ocular health and fetal or neonatal safety must be considered [57].

Many antiglaucoma medications carry potential fetal risks and have historically been classified according to FDA or WHO pregnancy risk categories. In clinical practice, the lowest effective dose should be used, and punctal occlusion is recommended to reduce systemic absorption. Laser or surgical interventions should be considered when medical therapy is ineffective or associated with unacceptable fetal risk. Selective laser trabeculoplasty (SLT) may represent a relatively safe and effective option during pregnancy, as it can reduce or eliminate the need for topical medications, although its efficacy may be variable and not always sustained. In cases of uncontrolled or advanced disease, trabeculectomy or implantation of glaucoma drainage devices may be performed under local anesthesia when clinically indicated. Table 9 below presents the particularities related to congenital and infantile glaucoma during pregnancy [8].

**Table 9:** Particularities related to congenital and infantile glaucoma during pregnancy

Appearance	Congenital glaucoma	Juvenile glaucoma
Onset	Birth–1 year	Teen/young adult
IOP changes in pregnancy	May decrease, but lifelong treatment often needed	May decrease slightly
Medication concerns	Same principles, but often already surgical	Same principles; adjust meds if pregnant
Surgery	Usually needed early in life; pregnancy rarely affects	Consider if uncontrolled medically
Follow-up	Multidisciplinary approach	Regular ophthalmic monitoring

## Discussion

The management of glaucoma during pregnancy continues to evolve, driven by technological advancements, a deeper understanding of gestational ocular physiology, and a growing body of evidence regarding the safety and efficacy of available treatments. Although current strategies–including medical therapy, laser interventions, and surgical procedures–provide a strong foundation for care, significant knowledge gaps remain. There is a continued need for high-quality research to refine therapeutic algorithms and further minimize risk to both mother and fetus. Future therapeutic strategies should prioritize interventions that achieve effective intraocular pressure (IOP) control while limiting systemic exposure.

### Emerging glaucoma medications and safety considerations

An important frontier in glaucoma management is the development of medications with improved safety profiles for use during pregnancy. Given the limited clinical data on existing therapies, therapeutic decision-making remains complex and challenging [4, 17, 58]. Expanding our understanding of the teratogenic risks associated with current drugs, along with the development of formulations that exhibit favorable pharmacokinetic and pharmacodynamic properties during pregnancy, holds significant promise for improving outcomes 60] [42].

Future research is likely to focus on the development of selective carbonic anhydrase inhibitors (CAIs) and prostaglandin analogs designed to minimize systemic absorption and, consequently, fetal exposure [59, 60]. Innovations in nano formulations and targeted drug delivery systems offer additional potential, enabling highly localized treatment with minimal systemic effects. Ultimately, the goal is to develop therapies that not only provide effective IOP control but are also demonstrably safe for use during pregnancy. Intracameral or intravitreal biodegradable extended-release implants are under evaluation. Currently, the bimatoprost extended-release implant, approved by the FDA in 2020, is the first pro-posed intracameral biodegradable extended-release implant for IOP reduction in patients with POAG or OHT, to be marketed as Durysta (Allergan) [18].

### Advances in laser therapies

Selective laser trabeculoplasty (SLT) has already become a mainstay in the management of glaucoma during pregnancy. Nevertheless, ongoing innovations in laser technology continue to expand therapeutic possibilities. Emerging procedures and refinements of established techniques aim to achieve more precise and effective intraocular pressure (IOP) reduction while minimizing risks to both maternal and fetal health [18].

Micro-invasive laser modalities, such as laser suture lysis and laser cyclophotocoagulations, represent promising alternatives for patients with advanced or treatment-resistant diseases. Additionally, combining laser therapy with limited pharmacological agents may enhance long-term disease control while reducing the need for systemic medications [18].

Further research is needed to determine optimal procedural timing, laser parameters, and safety protocols specifically for pregnant patients. A clearer understanding of how pregnancy-related hormonal and vascular changes influence laser outcomes will be critical for developing individualized treatment strategies. Advances in imaging technologies, such as enhanced depth imaging optical coherence tomography (EDI-OCT), will further support real-time monitoring and personalized care.

### Progress in minimally invasive surgical procedures

Minimally invasive glaucoma surgeries (MIGS) represent a rapidly expanding area in the surgical management of glaucoma during pregnancy. Compared to traditional filtering surgeries, MIGS techniques involve smaller incisions, shorter recovery times, and a lower risk of complications–features that are particularly valuable in pregnant patients [4, 12, 14, 29].

Devices such as the iStent, Hydrus Microstent, and Xen Gel Stent have shown encouraging outcomes; however, further research is needed to confirm their long-term safety and efficacy in pregnant populations. Of particular interest is the advancement of conjunctival-sparing MIGS techniques [52, which aim to preserve conjunctival integrity and reduce the risk of postoperative complications [18].

Minimally invasive conjunctival surgeries (MICS) further expands this paradigm by eliminating the need for conjunctival incisions, promoting bleb-independent IOP control, and potentially offering safer surgical options for pregnant patients. In addition, advances in minimally invasive cyclophotocoagulation–which targets aqueous humor production–may provide alternative solutions in refractory glaucoma [18].

In general, these procedures require the use of antimetabolites, which are considered the standard of care in subconjunctival filtration surgery. Examples include trabeculectomy, the Ex-PRESS glaucoma filtration device (Alcon, Fort Worth, TX, USA), the XEN45 microstent (Allergan, Dublin, Ireland), and the PRESERFLO™ MicroShunt (Santen Pharmaceutical Co., Osaka, Japan). The adjunctive use of antimetabolites has been shown to significantly reduce the risk of surgical failure by limiting postoperative fibrosis.

Despite these promising developments, large-scale, well-designed studies are essential to validate the safety and efficacy of MIGS and MICS procedures during pregnancy.

### Telemedicine and remote monitoring innovations

The integration of telemedicine and remote monitoring technologies presents a promising avenue for enhancing glaucoma management during pregnancy. Given the logistical challenges often associated with frequent in-person visits during gestation, tele-medicine offers a valuable means of maintaining consistent and accessible care.

Remote monitoring devices, such as home tonometers and digital imaging systems, allow for regular tracking of intraocular pressure and ocular health from the comfort of the patient’s home, thereby reducing the need for travel and in-clinic appointments [4, 18]

Additionally, telemedicine platforms enable timely communication between patients and healthcare providers, promote medication adherence, and support rapid clinical decision-making. As digital health infrastructure continues to expand, its role in patient education, symptom monitoring, and longitudinal surveillance is expected to become increasingly relevant in managing chronic conditions such as glaucoma during pregnancy [18].

### Personalized and precision medicine approaches

Advances in genetics and molecular biology are paving the way for personalized medicine approaches in glaucoma care. These may include genetic screening, ocular biomarker profiling, and individualized imaging strategies [18, 60]

A deeper understanding of the interaction between pregnancy-related hormonal fluctuations and glaucoma pathophysiology may facilitate development of targeted, pregnancy-adapted treatment strategies. As precision medicine evolves, clinicians may become better equipped to predict disease progression, optimize therapeutic responses, and tailor interventions to individual maternal and fetal considerations. [60].

### Strengthening multidisciplinary care models

Effective glaucoma management during pregnancy requires close multidisciplinary collaboration among ophthalmologists, obstetricians, maternal–fetal medicine specialists, and anesthesiologists. Integrated care models support coordinated decision-making, optimize risk management, and ensure comprehensive attention to both ocular and systemic health [7, 18, 61].

During the preconception period, the ophthalmologist plays a crucial role in evaluating and counseling patients with known ocular hypertension or glaucoma. This evaluation includes assessing the degree of ocular damage, determining the target intraocular pressure, and selecting the most appropriate therapeutic options both be-fore conception and throughout pregnancy.

In cases where treatment is absolutely necessary, the patient should be informed that there are safe therapeutic alternatives, such as certain laser procedures.

Moreover, interdisciplinary communication is essential. The ophthalmologist should ensure that both the obstetrician and pediatrician are adequately informed, so that coordinated and specialized monitoring of the patient can be maintained during pregnancy and in the postpartum period.

Furthermore, such collaborative approaches enhance patient education and empower pregnant women to actively engage in shared decision-making processes about their treatment and care. In managing complex conditions like glaucoma during pregnancy, a multi-disciplinary approach is essential to achieving the best possible outcomes for both mother and child [18, 49].

### 4.7. Research limitations

This review has several limitations. Firstly, although a comprehensive literature search was performed, the number of studies meeting inclusion criteria remains relatively limited. Secondly, the heterogeneity of the included studies limited the feasibility of a consistent and comparative analysis. Furthermore, the number of original experimental studies was notably low–an anticipated limitation, given that such research can only be ethically conducted using animal models.

In the absence of evidence-based data, the teratogenic potential of most therapeutic agents in pregnant and lactating women remains largely unknown. In general, none of the available IOP-lowering drugs can be considered completely safe during pregnancy and lactation.

## Conclusions

The future of glaucoma management during pregnancy is promising and rapidly evolving. Advances in pharmacologic therapies, innovative laser and surgical techniques, telemedicine integration, and precision medicine are poised to further refine patient care. Ongoing research and interdisciplinary collaboration will be essential in developing new approaches while safeguarding both maternal and fetal health.

As a recommendation, preconception is the ideal period to optimize drug therapy or to propose other therapeutic options, such as laser or MIGS, with the aim of achieving good IOP control before pregnancy. All medications should be avoided whenever possible during the first trimester, i.e. during organogenesis, and used cautiously in late pregnancy, since they can cross the placenta and affect the fetal cardiovascular, respiratory and neurological function.

As innovation progresses, clinicians will be better equipped to deliver safe, personalized, and effective glaucoma care for pregnant patients, ultimately improving outcomes for this unique and often challenging population.

## Data Availability

The datasets used and analyzed during the current study are available from the corresponding author upon reasonable request.
